# Replication competent HIV-1 viruses that express intragenomic microRNA reveal discrete RNA-interference mechanisms that affect viral replication

**DOI:** 10.1186/2045-3701-1-38

**Published:** 2011-11-23

**Authors:** Zachary Klase, Laurent Houzet, Kuan-Teh Jeang

**Affiliations:** 1 Molecular Virology Section, Laboratory of Molecular Microbiology, National Institute of Allergy and Infectious Diseases, Bethesda MD, 20892, USA

**Keywords:** miRNA, HIV-1, RNA interference, viral replication, miR326, miR211

## Abstract

**Background:**

It remains unclear whether retroviruses can encode and express an intragenomic microRNA (miRNA). Some have suggested that processing by the Drosha and Dicer enzymes might preclude the viability of a replicating retroviral RNA genome that contains a *cis*-embedded miRNA. To date, while many studies have shown that lentiviral vectors containing miRNAs can transduce mammalian cells and express the inserted miRNA efficiently, no study has examined the impact on the replication of a lentivirus such as HIV-1 after the deliberate intragenomic insertion of a *bona fide *miRNA.

**Results:**

We have constructed several HIV-1 molecular clones, each containing a discrete cellular miRNA positioned in *Nef*. These retroviral genomes express the inserted miRNA and are generally replication competent in T-cells. The inserted intragenomic miRNA was observed to elicit two different consequences for HIV-1 replication. First, the expression of miRNAs with predicted target sequences in the HIV-1 genome was found to reduce viral replication. Second, in one case, where an inserted miRNA was unusually well-processed by Drosha, this processing event inhibited viral replication.

**Conclusion:**

This is the first study to examine in detail the replication competence of HIV-1 genomes that express *cis*-embedded miRNAs. The results indicate that a replication competent retroviral genome is not precluded from encoding and expressing a viral miRNA.

## Background

RNA interference (RNAi) is a regulatory mechanism conserved in organisms from protozoans to mammals [1-3]. This process employs a small single stranded RNA of 20-24 nucleotides in length which is used as a guide-RNA to direct an RNA-induced silencing complex (RISC) containing the argonaut protein and co-factors to the targeted RNA [4-8]. Human cells encode 1,527 miRNA genes [9] that are transcribed into precursor primary miRNAs (pri-miRNAs) which are processed in the nucleus by Drosha into shorter hairpin products called pre-miRNA. The pre-miRNAs are exported into the cytoplasm by Exportin-5, and cleaved by Dicer to generate 20-24 nucleotide RNA duplexes, one strand of which is loaded into the Argonaute containing RISC [10-14]. miRNA-RISC complexes can silence target mRNAs via imperfect complementarity with sequences located in the 5'-UTR [15-17], coding sequences [18,19], and most commonly the 3'-UTR [2,20,21].

The RNAi pathway is pleotropically functional in many diverse biological processes; and its dysregulation leads to a plethora of pathologies including cancers, metabolic disorders, and infectious diseases [22-24]. In plants, RNAi as a host defense against viral infections has been well-established [25-27]. In vetebrates, the efficacy of RNAi based antiviral defense is debated [28-32], although several findings support the importance of this mechanism [33-40]. Additionally, there is substantial evidence that RNAi is employed by cells as a mean to keep mammalian endogenous retroviruses (i.e. retrotransposons) under check [41-46].

Several studies have reported that cellular miRNAs can modulate HIV-1 replication in human cells. For example, it was found that miR-28, miR-125b, miR-150, miR-223 and miR-382 function to induce HIV-1 latency in T-cells [47], and that these same miRNAs conferred resistance to HIV-1 infection in blood monocytes [48]. Furthermore, miR-29 has been shown to silence HIV-1 mRNAs that contain *Nef *sequences [49-51]. Finally, there is accumulating evidence that some cellular miRNAs may indirectly affect HIV-1 through the regulation of cellular proteins, such as Cyclin T1 and PCAF, which are employed for viral replication [52,53]. These findings are underscored by studies demonstrating, in models of spreading infection, that the over-expression of proteins involved in RNAi decreases viral replication while the knock-down of these proteins increases viral replication [52,54-56]

Relevant to their interaction with host cells is the question whether retroviruses can encode viral miRNAs. Some have suggested that the potential vulnerability of RNA-genomes to processing by RNAse III enzymes such as Drosha and Dicer might preclude RNA-viruses from containing *cis*-embedded miRNAs [37,57-59]. However, it has been shown that the infecting retroviral genome is apparently shielded by RNA-binding proteins rendering it inaccessible to targeting by RNAi factors [60]. Thus, it remains an open question whether a replication competent retroviral genome can encode a viral miRNA. Relevant to this issue, multiple laboratories have reported the processing of the viral TAR RNA into a miRNA-like non-coding RNA in HIV-1 infected cells [61-65]. The complexity of this multitude of findings cautions that a full understanding of the functions of HIV-1 associated non-coding RNAs awaits further investigation [64].

The current study was undertaken to answer more clearly whether an HIV-1 genome encoding an intragenomic miRNA is precluded from replication competence. We approached this question by creating several HIV-1 molecular genomes that contain discrete cellular miRNAs positioned in the *Nef *gene. We asked whether the intragenomic presence of the inserted miRNA in HIV-1 prevents viral replication in human cells. Our results showed no absolute preclusion in human cells against the replication of an HIV-1 genome expressing an intragenomic miRNA.

## Results

### Construction of five discrete HIV-1 molecular clones containing intragenomic miRNA

**Figure 1:**
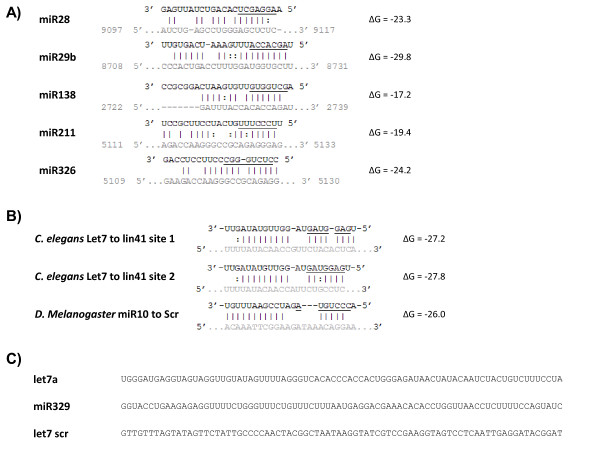
**Sequences of miRNAs and their predicted RNA targets**. **(A) **The putative target sites (with indicated ΔGs) for miR28, miR29b, miR138, miR211 and miR326 in the HIV-1 genome are shown. The miRNA sequence is listed at the top, with the seed sequence underlined; the HIV-1 target sequence is listed at the bottom in the 5' to 3' orientation. The numbers indicate the target position in the HIV NDK proviral genome. **B) **Three examples of published miRNA-mRNA target pairs (with ΔG values) with indicated mismatches or G:U pairings in the seed sequence. **C) **The primary miRNA sequences of let7a, miR329 and let7 scr which were inserted into the pNL4-3 proviral plasmid.

**Figure 2:**
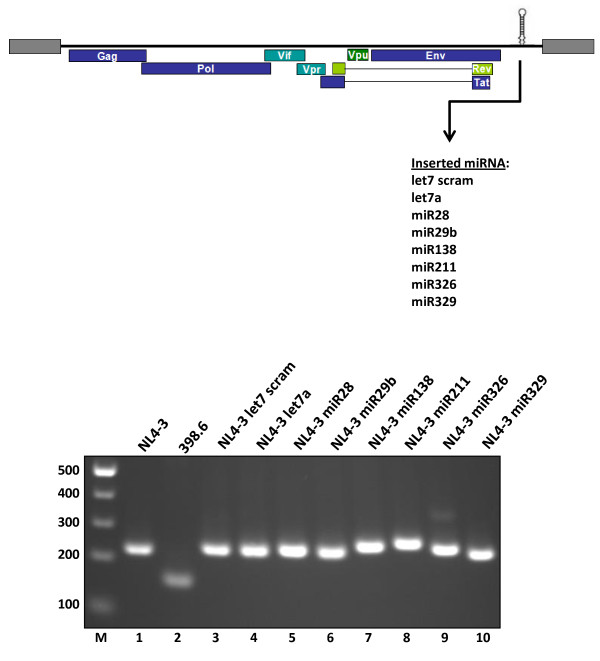
**Cloning of cellular miRNA sequences into NL4-3**. Schematic representation (Top) of the NL4-3 genome showing the insertion of the miRNA sequences into the *Nef *locus. The bottom panel shows PCR analyses of the indicated molecular clones performed to verify insertion. The p398.6 clone was a shuttle vector used in transferring the cloned miRNA into the pNL4-3 full length molecular clone. PCR primers were selected that flank the insertion site in *Nef*. Wild-type virus (lane 1) yielded a band of 220 bp. The p398.6 shuttle vector (lane 2) yielded a band of 143 bp. Insertion of the miRNA sequences (lanes 3-10) yielded a PCR fragment size commensurate with the size of the insert. Sizes of the molecular weight ladder (M) are indicated on the left.

We began our study by individually cloning five cellular miRNAs separately into the *Nef *locus of the NL4-3 molecular clone (Figure 1A) [66]. This locus was chosen because it is well-established that the HIV-1 *Nef *gene is dispensible for viral replication in cultured human cells [66,67] The five cellular miRNAs, miR28, miR29b, miR138, miR211, and miR326, were selected because each potentially has a complementary target sequence in the HIV-1 genome (Figure 1A). Figure 1A illustrates the imperfect complementarities, with the respective calculated free energies for duplex formation, between the various miRNAs and their putative HIV-1 target sequences. For comparison purposes, several cellular miRNA - mRNA pairs with similarly imperfect complementarities that have been reported in the literature are shown in Figure 1B[68-70]. We also cloned two additional miRNAs, let7a and miR329, into HIV-1 NL4-3. Based on target site prediction, these two miRNAs have no target sequence complementarities in the HIV NL4-3 genome. A final clone was created to incorporate a scrambled version of the let7a sequence. This sequence, designated as let7 scr, cannot fold into an RNA hairpin and is not expected to be processed into a miRNA (Figure 1C). All the chimeric NL4-3-miRNA genomes were checked by restriction digestion to verify a correctly-sized insert (Figure 2), and each clone was directly sequenced to confirm the expected identity.

### Expression of the inserted miRNA from the chimeric NL4-3 miR molecular clones

**Figure 3:**
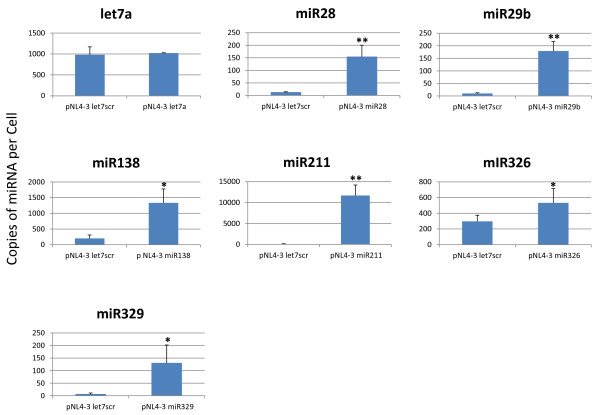
**NL4-3 miR viruses express miRNA in human cells**. 293T cells were seeded in a 6-well plate and transfected with 2 μg pNL4-3 let7 scr, pNL4-3 let7a, pNL4-3 miR28, pNL4-3 miR29b, pNL4-3 miR138, pNL4-3 miR211, pNL4-3 miR326 and pNL4-3 miR329. RNA was extracted at 48 hours post transfection and used to determine miRNA levels using qPCR. Copy number per cell was determined based on normalization to miR16 and the assumption of 10 pg of RNA per cell. Each graph shows the expression of a specific miRNA after transfection of the indicated chimeric NL4-3 molecular clone. * indicates a p-value < 0.05 and ** indicates a p-value < 0.01.

We evaluated the expression of the inserted miRNAs from the NL4-3-miRNA clones. 293T cells were transfected individually with two micrograms of pNL4-3 let7 scr, pNL4-3 let7a, pNL4-3 miR28, pNL4-3 miR29b, pNL4-3 miR138, pNL4-3 miR211, pNL4-3 miR326, or pNL4-3 miR329. Forty-eight hours after transfection, RNA was extracted from the cells, and miRNA levels were quantified using the QuantiMiR qPCR kit (Systems Biosciences). The copy numbers per cell of let7a, miR28, miR29b, miR138, miR211, miR326 and miR329 were determined based on 10 pg of RNA per cell (Figure 3). We found that the transfection of pNL4-3 miR28, pNL4-3 miR29b, pNL4-3 miR138, pNL4-3 miR326 and pNL4-3 miR329 increased the expression of the corresponding miRNAs by 142, 169, 1134, 236 and 124 copies per cell, respectively. The basal level of miR326 in 293T cells is relatively high at 297 copies per cell; however, the transfection of pNL4-3 miR326 into the cells further increased this amount by 236 copies. The transfection of pNL4-3 miR28 and pNL4-3 miR29b increased the expression of the corresponding miRNAs by 142 and 169 copies, respectively. The transfection of pNL4-3 miR211 increased miR211 copy number by 11,597 per cell (a greater than 100 fold increase). The transfection of pNL4-3 let7a produced the smallest increase in copy number adding only 40 additional copies per cell. Despite individual variabilities, the overall results support that most miRNAs cloned into HIV-1 *Nef *are expressed on the order of 10^2 ^copies or more per cell, with the exception of miR211 which is expressed at a considerably higher level.

### Intragenomic expression of miR28, miR211 and miR326 reduced single cycle HIV-1 infectivity

**Figure 4:**
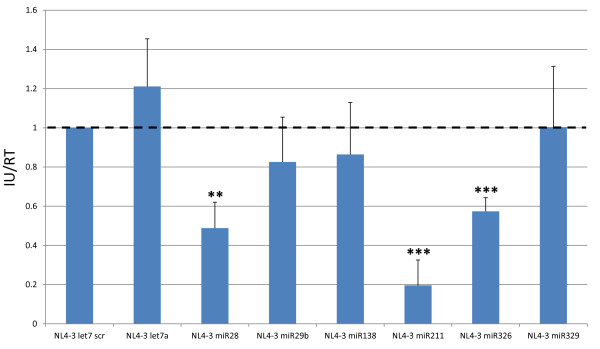
**Analyses of viral stocks generated from transfection in 293T cells**. 293T cells were seeded in a 6-well plate and transfected with 2 μg pNL4-3 let7 scr, pNL4-3 let7a, pNL4-3 miR28, pNL4-3 miR29b, pNL4-3 miR138, pNL4-3 miR211, pNL4-3 miR326 and pNL4-3 miR329. Supernatants were harvested at 48 hours post transfection and filtered to remove any contaminating cells. Supernatants were used to infect the HeLa derived TZMbl reporter cell line and to determine reverse transcriptase (RT) activity. Twenty-four hours after infection, the TZMBl cells were fixed and infected cells were visualized by β-galactosidase staining. Infectious units (IU)/RT were determined and graphed relative to NL4-3 let7 scr control. Data shown are the averages of three replicates. ** indicates a p-value < 0.01 and *** indicates a p-value < 0.001 compared to control.

We next determined if expression of the intragenomic miRNA influences HIV-1 replication. We examined the number of infectious HIV-1 virions produced from transfection into cells of the respective NL4-3-miRNA genomes. Accordingly, 293T cells were transfected individually with two micrograms of pNL4-3 let7 scr, pNL4-3 let7a, pNL4-3 miR28, pNL4-3 miR29b, pNL4-3 miR138, pNL4-3 miR211, pNL4-3 miR326 or pNL4-3 miR329. Forty-eight hours after transfection, supernatants were harvested, assayed for reverse transcriptase activity, and equal RT-amounts were used to infect TZMbl indicator cells [71]. Twenty-four hours after infection, the TZMbl cells were fixed, and the infectivity of the respective samples was determined by β-galactosidase assay. The relative infectivity of each sample was measured by counting the number of blue cells and comparing the value relative to the number produced from infection with the NL4-3 let7 scr virus (Figure 4). Compared to the NL4-3 let7 scr control, no significant difference was seen for the NL4-3 let7a, NL4-3 miR329, NL4-3 miR29b, and NL4-3 miR183 viruses; however, viruses expressing miR28, miR211 and miR326 showed a statistically significant reduction in infectivity (by 49%, 20% and 57%, respectively). The absence of significantly down-regulated replication for the miR29b expressing virus is puzzling because miR29b has been shown previously by others to reduce HIV-1 gene expression [49,50]. It is possible that different levels of miRNA expression and/or cell type or cell culturing differences account for the divergent experimental results.

### Reduced infectivity of NL4-3 miR211 and NL4-3 miR326 arises from different mechanisms

The NL4-3 miR21, the NL4-3 miR326 and the NL4-3 miR28 viruses displayed reduced infectivity. We noted that the NL4-3 miR211 virus expressed its corresponding miRNA at a much higher level (>11,000 copies of miR211 per cell) than the NL4-3 miR326 virus (Figure 3). It is possible that an overly efficient processing of a *cis*-embedded miRNA within a viral RNA genome may deleteriously affect viral replication. Potentially, the reduced replication of the NL4-3 miR211 virus may be explained by this mechanism. By contrast the reduced replication of the NL4-3 miR326 virus may be because the miR326 expressed from *Nef *led to the silencing of its complementary NL4-3 target sequence (Figure 1A).

**Figure 5:**
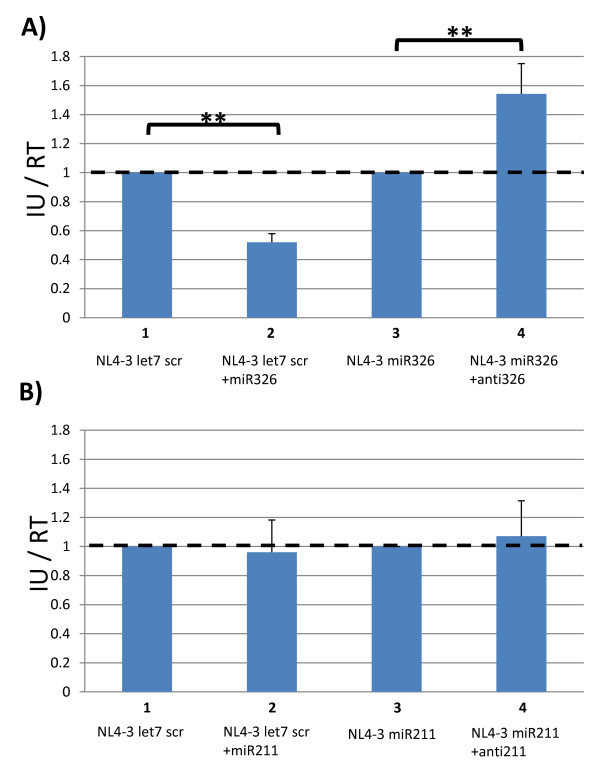
**Analyses of the *trans *expression of miRNAs and antagomirs on the infectivity of the indicated NL4-3 viruses**. 293T cells were seeded in a 6-well plate and transfected with 2 μg of proviral plasmid and 100 pM of the indicated RNA. Supernatants were harvested at 24 hours post transfection, filtered and used to determine infectious units as normalized by RT activity. (**A) **Evaluation of NL4-3 miR326 virus. NL4-3 let7 scr was co-transfected with either control RNA (column 1) or miR326 (column 2). NL4-3 miR326 was co-transfected with either control RNA (column 3) or an anti-miR326 antagomir (column 4). Results are shown relative to the control NL4-3 let7 scr virus. (**B) **Evaluation of NL4-3 miR211 virus. NL4-3 let7 scr was co-transfected with either control RNA (column 1) or miR211 (column 2). NL4-3 miR211 was co-transfected with either control RNA (column 3) or an anti-miR211 antagomir (column 4). Results are shown relative to the control NL4-3 let7 scr virus. All data are the averages of three replicates. ** indicates a p-value < 0.01.

To investigate the above possibilities, we employed synthetic miRNA-mimics or anti-miRNAs (antagomir) to effect or obstruct miRNA-mediated silencing. Thus, 293T cells were transfected with pNL4-3 let7 scr molecular clone with or without 100 pM synthetic miR326-mimic; and separately 293T cells were transfected with pNL4-3 miR326 with or without 100 pM synthetic anti-miR326 antagomir. Cell culture supernatants were harvested from these transfections, filtered, and measured for infectious units via infection of TZMbl cells (Figure 5A). We observed that transfected pNL4-3 let7 scr in the setting of transfected synthetic miR326, but not control miRNA mimic, produced a lowered viral infectivity. By contrast, pNL4-3 miR326 in the setting of synthetic miR326 antagomir showed an increased infectivity. These results are compatible with the interpretation that the reduction of NL4-3 miR326 replication is explained likely by miR326-mediated silencing of a complementary viral RNA target sequence.

We performed a similar analysis using the NL4-3 miR211 virus. In these experiments, 293T cells were transfected with pNL4-3 let7 scr with or without 100 pM of synthetic miR211, and pNL4-3 miR211 was separately transfected into 293T cells with or without 100 pM anti-miR211 antagomir. Next, cell culture supernatants were harvested and measured for infectivity using TZMbl cells (Figure 5B). Interestingly, the production of the NL4-3 let7 scr virus was not silenced by co-transfected synthetic miR211; nor was the infectivity of NL4-3 miR211 virus increased by co-transfected synthetic anti-miR211 antagomirs. These results suggest that the observed reduction in replication of the NL4-3 miR211 virus is unlikely due to miR211-mediated silencing of a putative complementary HIV-1 RNA target sequence (Figure 1A). Further experiments are needed to determine whether the predicted miR211 complementary viral sequence (Figure 1A) is not a competent target or if synthetic miR211 is not efficiently employed as a guide RNA by RISC.

**Figure 6:**
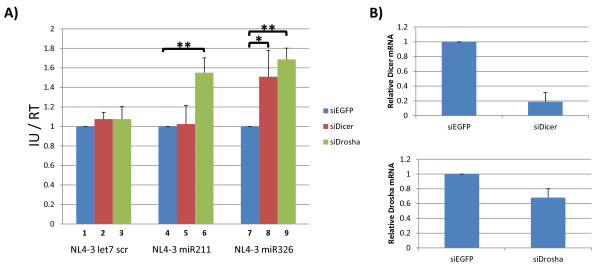
**The effect of Dicer and Drosha knockdown on NL4-3 miR326 and NL4-3 miR211 viruses**. 293T cells were seeded in a 6-well plate and transfected with 10 pM siRNA against EGFP (control), Dicer, or Drosha. Twenty-four hours post RNA transfection, the cells were transfected with 2 μg of pNL4-3 let7 scr, pNL4-3 miR326 or pNL4-3 miR211. (**A) **Supernatants were harvested 48 hours post viral transfection and used to determine infectious units as normalized to equal RT values. (**B) **Total RNA was extracted from transfected 293T cells and used to determine relative expression of Dicer (top panel) and Drosha (lower panel) by quantitative qPCR. Amounts were normalized to GAPDH. Data are shown relative to control (siEGFP) transfection with each virus and are the averages of three replicates. * indicates a p-value < 0.05 and ** indicates a p-value < 0.01.

The Drosha and Dicer proteins act sequentially inside the cell to process a precursor miRNA into a mature miRNA. It has been found that the knockdown of either protein affects the production of mature miRNAs. To ask if either Drosha or Dicer contributes to the reduced infectivity of the NL4-3 miR211 virus, we transfected 293T cells separately with siRNA against Dicer, Drosha, or EGFP, as a control. Twenty-four hours after the siRNA transfection, the cells were transfected with pNL4-3 let7 scr, pNL4-3 miR211, or pNL4-3 miR326. Forty-eight hours after the second transfection, cell culture supernatants were harvested, filtered and used to determine the number of infectious units using TZMbl cells (Figure 6A). The successful knockdown of Dicer or Drosha mRNA by the transfected siRNAs was confirmed by quantitative RT-PCR analyses of RNAs extracted from the indicated 293T cells (Figure 6B). We observed that neither the knockdown of Dicer nor Drosha produced appreciable effects on the infectivity of the NL4-3 let7 scr virus. Interestingly, the knockdown of Drosha increased the infectivity of the NL4-3 miR211 virus, while the knockdown of Dicer did not (Figure 6A, compare bar 4 to 5 and 6). In contrast, knockdown of either Dicer or Drosha increased the infectivity of NL4-3 miR326 (Figure 6A, compare bar 7 to 8 and 9). These results are compatible with the interpretation that the reduced replication of the NL4-3 miR211 virus may be due to overly robust nuclear processing by Drosha of viral mRNAs transcribed from the integrated pNL4-3 miR211 provirus. Indeed, qPCR analysis of the cell associated viral RNA confirmed a significant reduction of full-length viral RNA in the pNL4-3 miR211 transfected cells compared to pNL4-3 let7a or pNL4-3 miR326 transfected cells (Additional file 1). Because both Drosha and Dicer knockdowns are expected to reduce the production of mature miR326, the observed increase in the replication of the NL4-3 miR326 virus is consistent with a reduction of miR326-mediated mRNA silencing of this virus.

### Chimeric NL4-3 miR viruses produce a spreading viral infection in cultured cells

**Figure 7:**
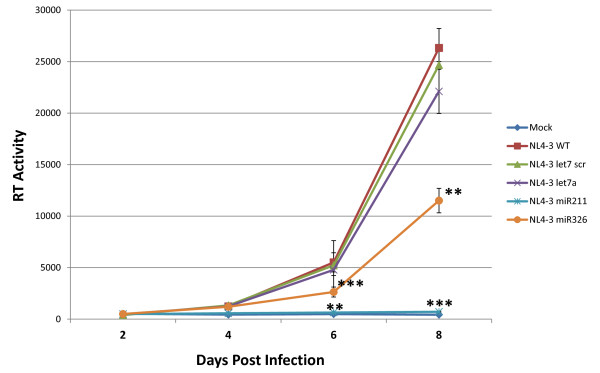
**The replication of chimeric NL4-3 miRNA viruses in cultured Jurkat T-cells**. Jurkat T-cells were infected with NL4-3, NL4-3 let7 scr, NL4-3 let7a, NL4-3 miR211 or NL4-3 miR326 as normalized by infectious units. Cell culture supernatants were sampled at days 2, 4, 6 and 8 post infection, and viral replication was determined by RT assay. Results shown are the averages of three independent infections per clone. * indicates a p-value < 0.01 and ** indicates a p-value < 0.01 as compared to NL4-3 let7 scr.

**Figure 8:**
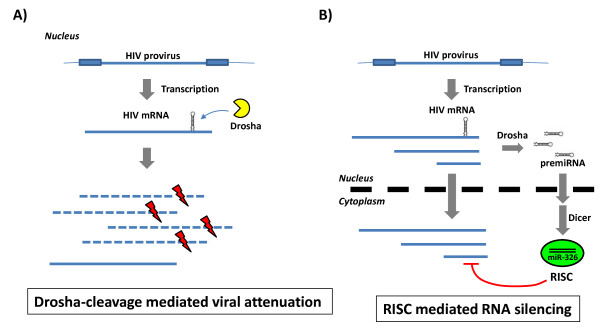
**Two mechanisms by which expressed intragenomic miRNAs can affect viral replication**. **(A) **An integrated HIV-1 genome is transcribed into viral mRNAs containing the embedded primary miRNA. This viral mRNA with an embedded primary miRNA may be recognized and cleaved in the nucleus by Drosha (in yellow). If Drosha-mediated cleavage is overly efficient, few uncleaved viral RNA would be left available to export into the cytoplasm for translation and/or packaging. This would then result in attenuated or incompetent viral replication like the NL4-3 miR211 virus. **(B) **For most chimeric NL4-3 miRNA viruses, Drosha processing of the embedded primary miRNA is relatively inefficient. Thus two pools of RNAs (unprocessed viral RNA and processed pre-miRNAs) are exported from the nucleus into the cytoplasm. In the case of the NL4-3 miR326 virus, the primary miR326 is further processed by Dicer into mature miR326 which associates with RISC and silences complementary viral target sequence leading to the attenuation of viral replication.

Next, we asked if chimeric NL4-3 miRNA viruses that express the inserted intragenomic miRNA support a spreading virus infection in cultured human cells. To answer this question, we generated stocks of NL4-3, NL4-3 let7 scr, NL4-3 let7a, NL4-3 miR211, and NL4-3 miR326 viruses to separately infect Jurkat T-cells (Figure 7). Wild-type NL4-3 virus, NL4-3 let7 scr virus, and NL4-3 let7a virus, all replicated in a spreading manner very similarly. By comparison, the NL4-3 miR326 virus also produced a spreading infection, albeit with a 50% reduction in virion levels as measured by RT on day 8. Interestingly, the NL4-3 miR211 virus was incompetent in promoting a spreading infection. These results indicate that the expression of an intragenomic miRNA within a retrovirus genome does not absolutely preclude replication competence. However, some miRNAs, like miR211, may be unusually good substrates for Drosha cleavage of the *cis*-inserted miRNA sequences resulting in an adverse outcome on virus replication. (Figure 8).

## Discussion

The ability of mammalian DNA viruses to encode viral miRNAs is well accepted. By contrast, it remains debated whether RNA viruses or retroviruses can encode and express viral miRNAs and remain replication competent. The current study tested the hypothesis that an HIV-1 genome with a *cis*-embedded miRNA can express the miRNA and propagate a spreading infection in cultured T cells.

To check this hypothesis, we constructed several chimeric HIV-1-miRNA molecular genomes with discrete cellular miRNAs cassetted into the viral *Nef *gene (Figure 2). For most of these chimeric genomes (NL4-3 miR28, NL4-3 miR29b, NL4-3 miR138, NL4-3 miR326 and NL4-3 miR329), the expression of the *Nef*-inserted miRNAs was several hundred copies per cell (Figure 3). One of the chimeric HIV-1 miRNA virus, NL4-3 miR211, had an unusually high level (>11,000 copies) of miRNA expression (Figure 3). When the viruses were tested for viral infectivity, three (NL4-3 miR28, NL4-3 miR211, and NL4-3 miR326) showed significantly reduced infectivity when compared to the NL4-3 let 7 scr and NL4-3 let7a control viruses (Figure 4).

The NL4-3 miR211 and NL4-3 miR326 viruses were studied in greater detail to understand the reason(s) for reduced infectivity. We explored two possible explanations. One possibility was that the expressed intragenomic miR211 or miR326 miRNAs recognize a *cis*-HIV-1 target sequence (Figure 1A) and that this miRNA-viral RNA interaction resulted in silencing, reducing viral infectivity. This explanation could be confirmed if an antagomir targeted against either miR211 or the miR326 would rescue the infectivity of the respective NL4-3 miR211 or pNL4-3 miR326 virus. That the infectivity of NL4-3 miR326, but not NL4-3 miR211, was rescued by a sequence-specific antagomir (Figure 5A) supported the interpretation that miRNA-viral RNA silencing explains the reduced infectivity of the former, but not the latter, virus.

What might explain the reduced infectivity of the NL4-3 miR211 virus? A second possibility is that the decreased infectivity may be due to unusually high efficiency of processing miR211 from NL4-3 transcripts that contain *cis*-inserted miR211 sequence. It may be that some miRNAs are simply better than other miRNAs as substrates for Drosha, Dicer, or both. If all viral RNAs with embedded-miR211 sequence were cleaved by Drosha or Dicer or both, then such events would severely hamper viral protein expression and could explain the severely attenuated infectivity of the NL4-3 miR211 virus. Indeed, we noted that the knockout of Drosha, but not Dicer, rescued NL4-3 miR211 infectivity (Figure 6A) while either knock down improved the infectivity of the NL4-3 miR326 virus (Figure 6A). Taken together with the findings in Figure 5, the results support that two different mechanisms are operative in reducing NL4-3 miR326 and NL4-3 miR211 viral replication (Figure 8).

Our NL4-3 miR211 virus results agree with a similar observation made by Liu *et al*. in their study of miRNA expression using a single round lentiviral gene delivery vector [57]. Liu *et al*. also found that the processing by Drosha of some miRNA-cassettes in lentivectors was one of several mechanisms that reduced particle titers. Because Drosha processing is a nuclear event, the likely scenario for reduced NL4-3 miR211 infectivity is the overly robust cleavage of miR211-embedded HIV-1 RNAs transcribed from the integrated proviral DNA genome (Figure 8), not from the cytoplasmic cleavage of miR211-embedded HIV-1 RNA genome. This interpretation agrees with the earlier observation made by Berkhout and colleagues that the infecting lentiviral RNA genome is well-protected from RNAi-mediated silencing [60].

Finally, we observed that the NL4-3 let7a and the NL4-3 miR326 viruses are capable of a spreading infection in cultured Jurkat T-cells. These results are consistent with no absolute preclusion against a replicating retrovirus encoding and expressing an intragenomic miRNA. Previously, it has been suggested that the proclivity of DNA viruses to replicate in the nucleus and RNA viruses to replicate mostly in the cytoplasm might explain why the former and latter have varying capacity for encoding viral miRNAs. Since a large part of the retroviral life cycle takes place in the nucleus and the genomic retroviral RNA in the cytoplasm is shielded by RNA-binding proteins [60], these processes may explain why some retroviruses like HIV-1 do produce modest levels of processed non-coding viral RNAs [61,63,64]. Other retroviruses like BLV are suggested to potentially encode more non-coding viral RNAs [72]. A recent study reports the expression of a miRNA-like small RNA from the highly structured 3' UTR of West Nile Virus and found that this RNA is supportive of viral replication [73]. If true, this report would represent another example of a miRNA or miRNA-like RNA encoded by an RNA virus. These reports, together with our currently demonstrated replication competence of HIV-1 genomes expressing inserted cellular miRNAs, encourage additional investigation into the nuanced miRNA-encoding capabilities of DNA viruses, RNA viruses, and retroviruses.

## Methods

### Cell culture

293T and TZMbl cells were maintained in DMEM supplemented with L-glutamine, Penicillin/Streptomycin and 10% fetal bovine serum. For transfections, cells were split 24 hours prior to transfection into 6-well plates at 500,000 cells/well. Cells were transfected with lipofectamine LTX (Invitrogen) according to manufacturer's instructions. For production of viral stocks, the supernatant was harvested at 48 hours after transfection. For siRNA transfections, the cells were first transfected with siRNAs and then re-transfected 24 hours later with proviral plasmids. Jurkat T-cell line was maintained in RPMI supplemented with L-glutamine, Penicillin/Streptomycin and 10% fetal bovine serum.

### RNA isolation, qRT-PCR and miRNA measurement

RNA was extracted using Trizol reagent (Invitrogen) following manufacturer's protocol. For the determination of mRNA levels, 1 microgram of RNA was used to create cDNA using the SuperScript III First-Strand Synthesis kit (Invitrogen). Following reverse transcription, the samples were diluted 1:50, and 2.5 microliters were used for quantitative PCR in a BioRad CFX96 or CFX384 qPCR machine. All mRNA analyses were normalized to GAPDH. Nucleic acid amplification was tracked by SYBR Green method. For miRNA quantitation, 1 microgram of RNA was processed using QuantiMir (Systems Bioscience Inc.); the resulting tagged cDNA was quantified using miRNA specific primers via qPCR. All miRNA analyses were normalized to the cellular miRNA miR16.

### Infections, RT and TZMbl assay

For infection of Jurkat cells, 6 × 10^6 ^cells were seeded in 2 ml of media and exposed to the indicated dose of virus supernatant for 24 hours. Cells were then washed and seeded in 10 ml of fresh RPMI, and sampled over time. Replication was measured through use of the RT activity assay: 5 μl of supernatant were added to 50 μl of RT reaction cocktail (60 mM TrisHCl, 75 mM KCl, 5 mM MgCl_2_, 1.04 mM EDTA, 0.1 NP-40, 5 μg/ml polyA and 0.16 μg/ml oligo dT) and incubated for two hours at 37°C. The reaction mix was spotted on DEAE membrane, washed with SSC, and dried before counting. For TZMbl assay, cells were seeded in a 96 well plate at 15,000 cells/well for 24 hours. Medium was then replaced with fresh RPMI containing serial dilutions of viral supernatant. Twenty-four hours post infection, the cells were washed, fixed and assayed for the presence of β-galactosidase by X-gal enzymatic assay. Blue cells were counted, and the number of infectious units per volume was computed based on the dilution of infecting supernatant.

### Sequencing

Cellular DNA from Jurkat T-cells at 8 days post infection was extracted using Qiagen DNA easy kit. PCR for insertion sites was performed, and the resulting fragments were gel purified and cloned into Invitrogen's TopoTA cloning vector before being directly sequenced.

### Cloning

For cloning for miRNA into NL4-3, we followed a previously described procedure [66]. In brief, the sequence of each pre-miRNA was determined by consulting the miRBase for the human miRNA [74,75]. PCR primers were designed to amplify these sequences with the addition of *Sal*I (5' end) and *Xho*I (3' end) to each pre-miRNA. PCR products were cloned into TopoTA vector (Invitrogen) and excised with *Sal*I and *Xho*I. Pre-miRNA fragments were then inserted into the *Xho*I site of a ΔNef shuttle vector, screened for orientation and then moved into the full length pNL4-3 proviral vector to produce the NL4-3 miR clones. All clones were sequenced to verify the proper insertion of the pre-miRNA sequence.

### Primers and oligonucleotides for cloning

Primers for generation of *Sal*I/*Xho*I miRNA precursors - let7a ATCGTCGACTGGGATGAGGTAGTAGGTTGTATAG/ACTCGAGTAGGAAAGACAGTAGATTGTATAG, miR28 ATCGTCGACGGTCCTTGCCCTCAAGGAGCTCACA/ACTCGAGAGTGCCTGCCCTCCAGGAGCTCACA, miR29b ATCGTCGACCUUCAGGAAGCUGGUUUCAUAUGGU/ACTCGAGCCCCCAAGAACACTGATTTCAAATG, miR138 ATCGTCGACCCCTGGCATGGTGTGGTGGGGCAGC/ACTCGAGCCTGTAGTGTGGTGTGGCCCTGGTG, miR211 ATCGTCGACUCACCUGGCCAUGUGACUUGUGGGC/ACTCGAGCTCCGTGCTGTGGGAAGTGACAACT, miR326 ATCGTCGACCTCATCTGTCTGTTGGGCTG/ACTCGAGTGAATCCGCCTCGGGGCTGG, miR329 ATCGTCGACGGTACCTGAAGAGAGGTTTTCTGGG/ACTCGAGGATACTGGAAAAGAGGTTAACCAGG

Oligonucleotides for the generation of let7 scr through annealing - ATCGTCGACGTTGTTTAGTATAGTTCTATTGCCCCAACTACGGCTAATAAGGTATCGTCC

GAAGGTAGTCCTCAATTGAGGATACGGATCTCGAGGCC, GGCCTCGAGATCCGTATCCTCAATTGAGGACTACCTTCGGACGATACCTTATTAGCCGTA

GTTGGGGCAATAGAACTATACTAAACAACGTCGACGAT

## Competing interests

The authors declare that they have no competing interests.

## Authors' contributions

ZK conceived of the study, participated in its design, performed the molecular analyses and wrote the manuscript. LH participated in the study design and assisted with the qPCR analysis. KTJ oversaw the work, the design, and the conception of the experiments and wrote the manuscript. All authors read and approved the final manuscript, and support was from NIAID intramural funding.

## Supplementary Material

Additional file 1 **Increased processing of viral genome-length RNAs in pNL4-3 miR211 transfected cells**. 293T cells were seeded in a 6-well plate and transfected with 2 μg pNL4-3 let7a, pNL4-3 miR211, or pNL4-3. Total RNA was extracted from the cells at 48 hours post transfection. RNA was treated with DNase, and cDNA was made by reverse transcriptase reaction using either poly dT (dT) or random hexamer (hexamer) as a primer. qPCR was performed on the cDNA to measure the presence of Gag RNA. In this assay, poly dT is anticipated to quantify genome-length Gag RNAs, while random hexamer will identify all genome-length as well as subgenome-length Gag RNAs. Quantities were normalized by GAPDH and shown relative to pNL4-3 let7a. * indicates a p-value < 0.01, ** indicates a p-value < 0.01, and *** indicates a p-value <0.001 as compared to NL4-3 let7a. Click here for file
