# Supplementary figures and images for: Replication competent HIV-1 viruses that express intragenomic microRNA reveal discrete RNA-interference mechanisms that affect viral replication
Source: Cell Biosci. 2011 Nov 23;1:38. doi: 10.1186/2045-3701-1-38 (PMC3256098; doi:10.1186/2045-3701-1-38)

Relative Gag RNA

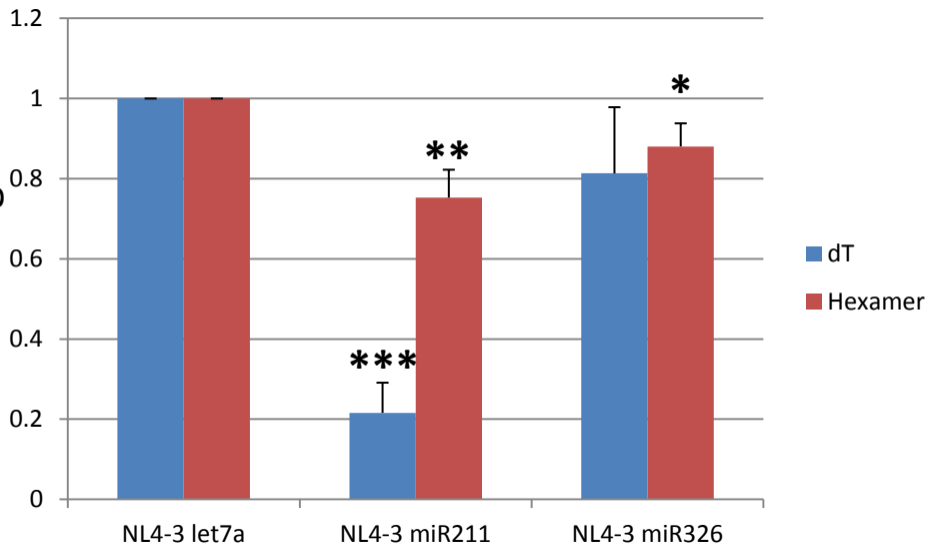

Supplement: Additional file 1 — Increased processing of viral genome-length RNAs in pNL4-3 miR211 transfected cells. 293T cells were seeded in a 6-well plate and transfected with 2 μg pNL4-3 let7a, pNL4-3 miR211, or pNL4-3. Total RNA was extracted from the cells at 48 hours post transfection. RNA was treated with DNase, and cDNA was made by reverse transcriptase reaction using either poly dT (dT) or random hexamer (hexamer) as a primer. qPCR was performed on the cDNA to measure the presence of Gag RNA. In this assay, poly dT is anticipated to quantify genome-length Gag RNAs, while random hexamer will identify all genome-length as well as subgenome-length Gag RNAs. Quantities were normalized by GAPDH and shown relative to pNL4-3 let7a. * indicates a p-value < 0.01, ** indicates a p-value < 0.01, and *** indicates a p-value <0.001 as compared to NL4-3 let7a. [file 2045-3701-1-38-S1.PDF]
